# Aggressive NK-cell leukemia in a 69 years old Caucasian woman: a case report

**DOI:** 10.1186/s40064-015-1553-y

**Published:** 2015-12-09

**Authors:** Sara Maj Hyldig Matzen, Klaus Kallenbach, Anne Regitze Reumert, Lars Munksgaard

**Affiliations:** Department of Clinical Biochemistry, Copenhagen University Hospital Roskilde, Koegevej 7-13, 4000 Roskilde, Denmark; Department of Clinical Pathology, Copenhagen University Hospital Roskilde, Koegevej 7-13, 4000 Roskilde, Denmark; Department of Hematology, Copenhagen University Hospital Roskilde, Koegevej 7-13, 4000 Roskilde, Denmark

## Abstract

Aggressive NK-cell leukemia is a rare malignancy mostly seen in younger Asians with a rapid clinical course and poor prognosis. Here, we describe a 69 years old Caucasian woman presenting with massive leukemization of neoplastic NK-cells. The cells were abnormal in morphology and surface marker expression and this clearly distinguished them from their normal counterpart. They were large and variable in shapes with irregular folding of the nuclei. By flow cytometry, their light scatter characteristics resembled normal monocytes. They showed bright expression of CD56 and CD2 but markedly decreased expression of CD7. They also expressed CD25. The patient presented with general malaise, including high fever, abdominal pain, signs and haemophagocytosis, and she quickly deteriorated and died 11 days after hospitalization. The origin of the leukemic cells of aggressive NK-cell leukemia is most likely the relatively scarce population of CD56^bright^ NK-cells, primarily residing lymph nodes and tonsils. The immunophenotype of the case presented here support this, adding CD25 expression which is not earlier addressed in this entity.

**Electronic supplementary material:**

The online version of this article (doi:10.1186/s40064-015-1553-y) contains supplementary material, which is available to authorized users.

## Background

The rare aggressive NK-cell leukemia (ANKL) is primarily described in Asian and South American populations (Lima 2013). The disease entity is described in the World Health Organization (WHO) classification for hematopoietic tumors and lymphoid tissues in 2008 but remains poorly characterized as the majority of publications comprise sporadic case reports. This especially holds true for European cases. The course of the disease is rapidly aggravating and nearly always fatal since the neoplastic cells respond poorly to therapy.

Here, we present a case story of an elderly Caucasian woman with an extremely short and aggressive disease course leaving no opportunities of treatment of her leukemia. The case was complicated by rapidly manifestation of serious enterococcus infection and general organ disability impact.

## Case description

**Fig. 1:**
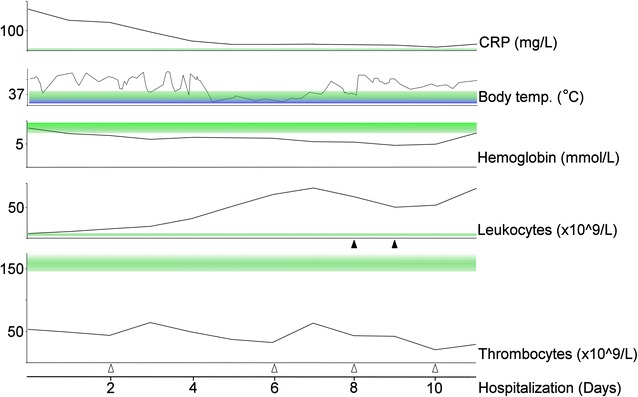
Laboratory indexes; Graphical presentation of the clinical parameters during the disease course. The CRP value decreased after the 1st days of treatment with corticosteroids and antibiotics, but it never reached normal level (<8 mg/L). Body temperature curve reveals a fluctuating picture that was only briefly and temporarily stabilized at non-feverish level. Haemoglobin was normal at admission but quickly decreased below normal range (7,3–9,5 mmol/L). Leukocyte count was also normal at admission but elevated during the 1st day and the leukocytosis persisted. Thrombocytes were markedly depressed during the entire hospitalization in spite of numerous transfusions, both thrombocyte transfusions (*open arrow heads*) and whole blood transfusions (*closed arrow heads*). Green bands indicate normal values for each of the parameters

A 69-year-old Caucasian female was admitted to the University Hospital, Roskilde, in August 2014, critically ill with high fever, septicaemi and suspected for acute leukemia. Two weeks earlier her husband reported, the patient admitted her general practitioner because of fever and started up with penicillin on a suspicion of pneumonia. After a few days she admitted the surgical department because of abdominal and lower back pain, and diarrhea. Medical history revealed a prior diagnosis of hypertension treated with two-drug therapy. A smoking history of more than 50 years was noted. Initially, more diagnoses came up and she was transferred to other hospitals twice (suspected for an aorta aneurism and cholecystitis). Initial laboratory assessments showed normal hemoglobin and leukocytes but thrombocytes were 53 × 10^9^/L (normal values are 145–390 × 10^9^/L). C-reactive protein (CRP) was markedly elevated to 210 mg/L (normal value <8 mg/L) and Lactate dehydrogenase (LDH) to 1770 U/L (normal values are 105–205 U/L). Leukocyte differential count gave suspicion of a leukemic state or secondary to serious infection. Flow cytometry confirmed a serious leukemic condition, and the patient was transferred to the regional hematological department. On the day of admission, leukocyte count rose above 80 × 10^9^/L (normal values are 3,5–8,8 × 10^9^/L), hemoglobin dropped to 5.4 mmol/L and need for on-going platelet transfusions to keep thrombocytes above 20 × 10^9^/L. Broad-spectrum antibiotics (meropenem) was immediately initiated, numerous blood tests, including blood cultures, viral antibodies and PCR were obtained. Intravenous steroid and rasburicase to prevent tumour lysis syndrome was started. Despite continuous antibiotic and steroid treatment the patients’ body temperature fluctuated at feverish level during most of the hospitalization period (see Fig. 1). Previous CT-scan had shown abdominal lymphadenopathy and splenomegali but no enlarged peripheral lymph nodes were visualized. From blood samples a first tentative diagnosis of T-LGL or NK-cell leukemia was made, but further immunophenotypic analyses were necessary to establish the exact diagnosis. Two days later, suddenly, the patients’ condition deteriorates and she had to be monitored at the intensive care unit. Creatinine was moderately elevated early in the disease course but now rose steadily along with stop in urine production. She needed assisted ventilation and medicine to maintain adequate blood pressure. The following day, the pathologist suggested a diagnosis of ANKL.

The patients’ condition allowed no active treatment for the leukemia other than steroid and supportive care and she died later that day, just 11 days after the admission to hospital. From blood cultures made on the day of demise a gram negative rod, Enterococcus faecium was found and later PCR for Epstein-Barr virus was positive (7200 copies/mL). This allowed a final diagnosis of ANKL as suspected. The patient’s blood was tested negative for anti-HIV 1-2, anti-HBc, anti-HCV antibodies, and HBsAg. The clinical picture and blood tests showed signs of haemophagocytosis.

See Fig. 1 for graphical presentation of development in laboratory parameters and Table 1 for an overview of key clinical features.

**Table 1:** Clinical data; comparison of clinical features usually presented in patients with ANKL and the clinical features presented in the patient in our case

	Classic clinical features of ANKL^a,b,c^	Clinical features of the case patient
Age (years)	30–50	69
EBV in leukemic cells	+	+
Symptoms at admission		
Fever	+	+
Lymphadenopathy	±	–
Splenomegaly	+	+
Hepatomegaly	±	–
LDH	Markedly increased	Markedly increased
Thrombocytopeni	+	+
Hemophagocytic syndrome	+	+

^a^(Lima 2013)

^b^(Lima et al. 2015)

^c^(Zhang et al. 2014)

### Morphology

**Fig. 2:**
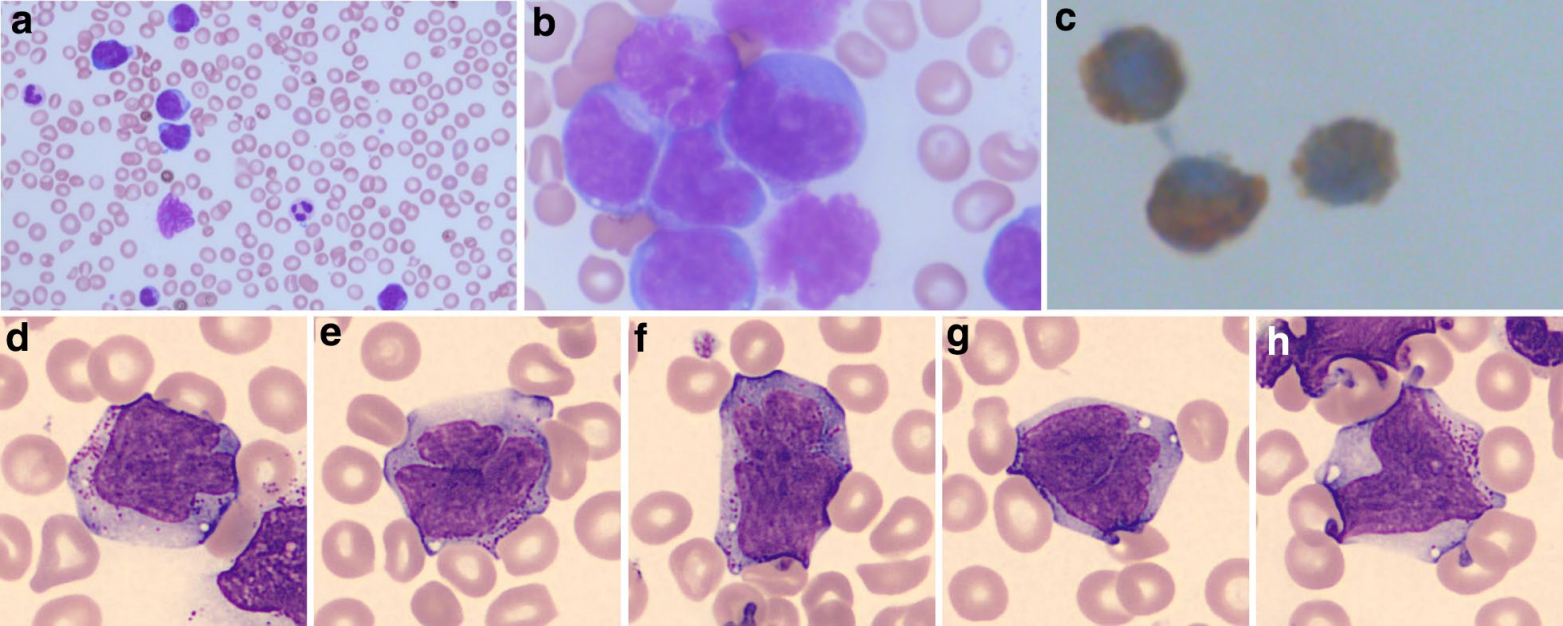
Morphologic features on peripheral blood smear. **a** (200x magnification) + **b** (630x magnification) May-Grünwald-Giemsa stain showing atypical cells with large, irregular folded nuclei with distinct nucleoli. The cytoplasm is basophilic with varying amount of fine granules. **c** CD3 immunostaining reveals a cytoplasmic reaction. **d**–**h** Representative images of the neoplasmic cells from CellaVision^®^ showing irregular nuclei and cytoplasmic granulation

A few peripheral blood samples were rendered for morphological review. They revealed prominent leukocytosis with approximately 90 % of the nucleated cells showing a polymorphic cell population with variation in size and shape (see Fig. 2a). The nuclei were large with irregular foldings and variably distinct nucleoli (see Fig. 2b, d–h). The surrounding cytoplasm was lightly basophilic containing dispersed azurophilic granules (see Fig. 2a–b, d–h). Cytochemical staining of myeloperoxidase was negative in the cell population of interest (data not shown). Immunophenotypic evaluation showed expression of CD3 (see Fig. 2c) while TdT-staining was negative (data not shown).

### Flow cytometric profile

**Fig. 3:**
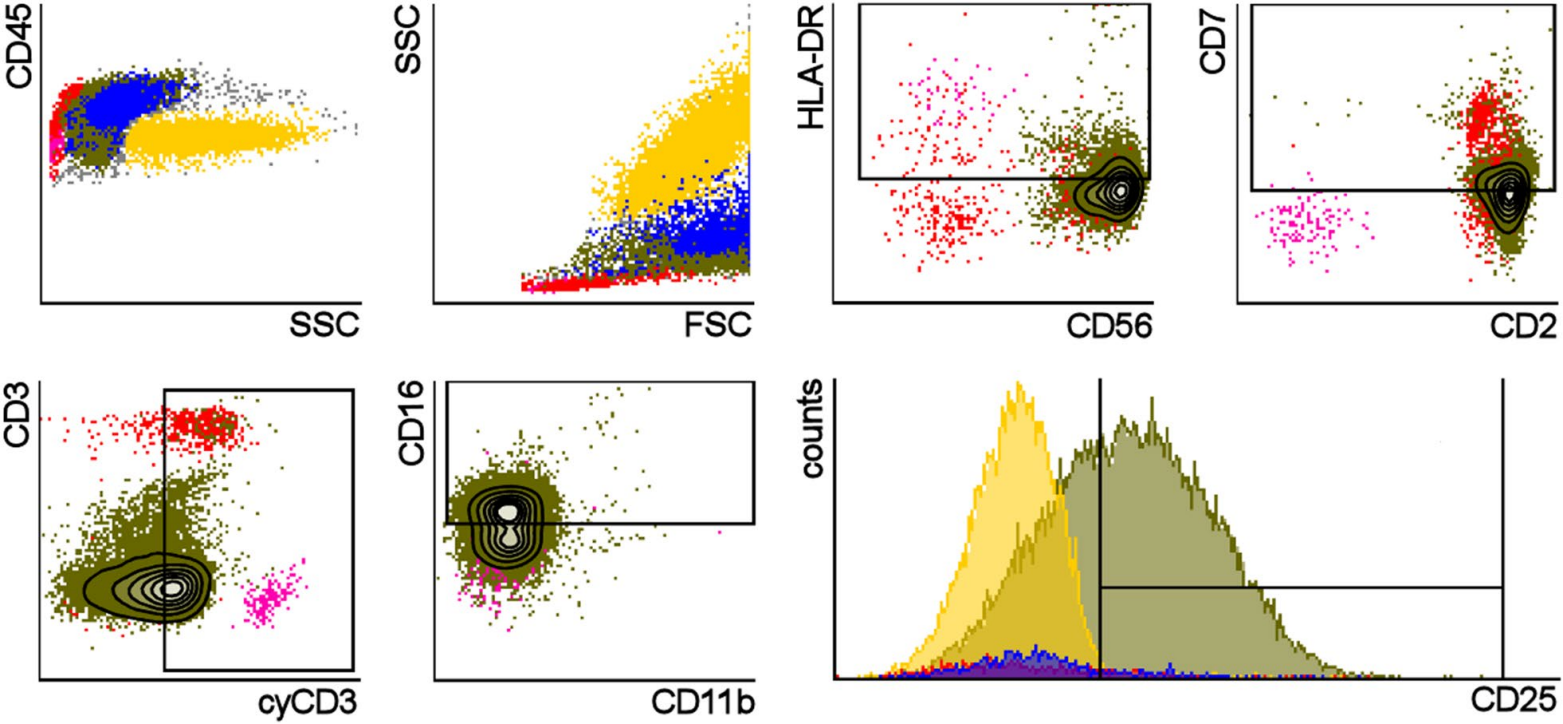
Flowcytometric data of peripheral blood; Neoplastic NK-cells (*green*) present in CD45/SSC-plot and in SSC/FSC-plot with characteristics similar to mature monocytes (*blue*). *Red and yellow dots* show lymphocytes and granulocytes, respectively. Remaining plots show specific staining for expression of relevant peptide markers. *Boxes* are placed around the positive portion of cell populations in cases where positive/negative discrimination is not obvious. Only 19 % of the leukemic population was positive for HLA-DR, whereas 42, 51, 52 and 63 % of the same population was positive for CD7, cyCD3, CD16 and CD25, respectively

A blood sample was sent for flow cytometric analysis. In order to determine the nature of the leukemic cells a wide range of antibodies was applied, of which most turned out negative. This included surface CD3, CD4, and CD8. The light scatter characteristics of the cells resembled that of mature monocytes, e.g. medium side scatter (SSC), high forward scatter (FSC). Bright expression of CD45, CD56 and CD2 lead the suspicion towards NK lineage and the concurrent dim expression of CD7, CD16 and cytoplasmic CD3ε consolidated the origin of the leukemia (see Fig. 3). The leukemic cells also expressed CD25. HLA-DR and CD11b are both reported to be occasionally expressed in this disease entity, but were negative in our case (for complete flowcytometric data, see Additional file 1: Figure S1).

Within the T-cell compartment, we found less than 2 % CD4-neg/CD8-neg, a hallmark of TCRγδ T-cells. This corresponded to <0,3  ‰ of the total nucleated cells in the sample.

### Molecular analysis of TCR gene rearrangements

**Fig. 4:**
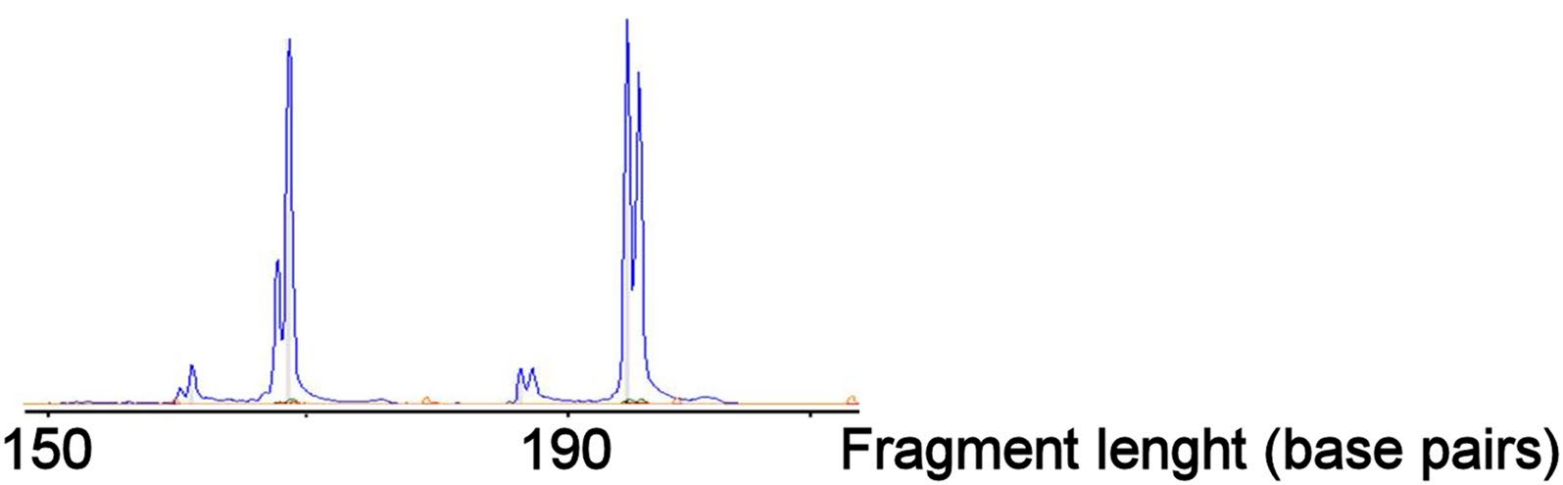
Molecular analysis of TCRdelta gene rearrangements in peripheral blood; Two prominent peaks are identified in fragment sizes 169 base pairs and 196 base pairs, respectively. The double-peak appearance is caused by DNA polymerase-catalyzed addition of nontemplated extra nucleotides

Peripheral blood DNA was subjected to molecular PCR-based analyses of TCR gene rearrangements using Biomed2 primers covering TCRgamma (TCRG), TCRbeta (TCRB) and TCRdelta (TCRD) genes. The analysis was performed in replicate. Polyclonal patterns were seen for TCRG and TCRB. Indication of clonal rearrangements was identified in TCRD gene, where two prominent peaks were identified (see Fig. 4). We suspect this to have a technical explanation. TCRD gene is removed when TCR alpha (TCRA) is rearranged and thus, only TCRγ/δ T-cells are certain to harbor rearranged TCRD. Therefore, the TCRD rearrangements will have inherent underrepresentation in a normal T-cell population potentially leading to paucity of TCRD templates for the PCR (Langerak et al. 2012). Since <0,3  ‰ of the total nucleated cells was assumed to be TCRγ/δ T-cells in this patient, it seems plausible that the clonal peaks identified in the peripheral blood are caused by preferential amplification and should be interpreted as pseudoclonality not associated to the malignant cell population.

## Discussion and evaluation

The recognition of the neoplastic cells was straightforward in the case presented here due to their massive dominance in peripheral blood. However, some patients present with only limited bone marrow or peripheral blood involvement despite heavy disease impact (Li et al. 2014). Flow cytometry is already incorporated as a standard screening tool in many hematologic settings. Since the patients at presentation often have symptoms suggestive of acute leukemia, the inclusion of NK-cell markers in the screening panel for acute leukemia would enable the detection of abnormal NK-cells even in cases with minimal infiltration. The increased size and granulation compared to normal peripheral NK cells places them in the monocyte gate when using CD45, SSC and FSC as segregation parameters. CD56 expression on monocytes is a well-known aberrancy in several diseases and is also seen on a small percentage of normal monocytes (Krasselt et al. 2013). Thus repudiating monocytic nature of the abnormal cells is prerequisite for recognition of the population. This could be done by including CD14 or CD64 in combination with CD56 in the screening panel.

Generally, NK cells are subdivided into two subpopulations based on their expression of CD56 and CD16. The CD56^bright^/CD16^neg/dim^ is normally a minority in peripheral blood but dominates in tonsils, lymph nodes and in deciduas during early pregnancy (Poli et al. 2009; Tao et al. 2015). They have limited cytotoxic capacity, but have been shown to be highly cytotoxic towards activated T-cells in comparison with the CD56^dim^ subset (Bielekova et al. 2006). It is hypothesized that neoplastic NK cells of ANKL represent a transformed counterpart of the CD56^bright^ subset (Lima et al. 2015). Since nearly all ANKL cases present with EBV DNA in the leukemic cells, it is tempting to assume that this transformation is initiated by the EBV infection. Supportive of the hypothesis is the fact that NK cells in lymph nodes have been identified as targets during early Epstein Barr virus infection (Trempat et al. 2002). A study of the immunophenotype of 29 de novo ANKL revealed bright expression of CD56 accompanied by lack of CD5- and CD57-ekspression as the only consistent pattern (Li et al. 2014). CD57 is only expressed in the normal CD56^dim^ compartment, supporting the theory of CD56^bright^ subset as the cell of origin in ANKL. However, more extensive subdivision of normal NK cells might be relevant. Fu, Tian and Wei suggest a fractioning of the CD56^bright^ subset into NK^regulatory^ and NK^tolerant^ by their expression or lack of CD27, respectively. The CD27^+^ regulatory NK cells are described as abundant cytokine producers (Fu et al. 2014). Uncontrolled cytokine production, often leading to hemophagocytic lymphohistiocytosis, is reported in many cases of ANKL (Akashi and Mizuno 2000). On that basis it is intriguing to suggest the NK^regulatory^ as probable cellular origin of ANKL. The endogenous immune evasive potential of a cell type naturally involved in modulation of immune response would provide supportive growth conditions for the malignant clone. However, to the best of our knowledge CD27-expression in ANKL has not been investigated and thus, the exact origin cannot be designated within the CD56^bright^ populations. It should be emphasized that the CD56^bright^ NK cells only recently gained wider investigational focus and that their biology and functions by no means are fully drawn up (Fu et al. 2014).

The majority of normal PB NK-cells are negative for CD25-expression and the same is true for NK-cells of Chronic NK-cell lymphoproliferative disorder (CLPD-NK) (Lima et al. 2004). To our knowledge, the marker has not been addressed in specific reports of ANKL. In a series-report of 12 patients with either ANKL or extranodal NK/T-cell lymphoma, nasal type (NKTCL) it was reported that 6 unspecified cases were tested negative for CD25 (Lima et al. 2015). In contrast, Yu and colleages found the marker consequently positive in a report on immunophenotype for NKTCL (Yu et al. 2014). Here, we report positive staining of the marker by flow cytometry with 63 % of the neoplastic cells showing a brighter signal than the negative granulocytes (see Fig. 3). CD25 expression is previously shown to be an indication of high proliferation potential in human NK-cells (Clausen et al. 2003). It is a key component of the high affinity heterotrimeric receptor for IL-2, a cytokine well-known for its properties in NK proliferation and enhancement of cytokine production (Bielekova et al. 2006). CD25-expression has been shown for decidual CD56^bright^ NK cells (Tao et al. 2015). It has also been shown that previously activated NK-cells are able to remember the activation and persist in the host with enhanced functional capacity. The authors designated this cell type cytokine induced memory-like (CIML) NK cells and hypothesize that the increased CD25 expression seen in these cells are at least partly responsible for their preferential expansion and reactivation in response to low dose IL2 [reviewed by (Romee et al. 2014)]. So far, one can only speculate to what extent the CD25 expression influence the course of the disease in ANKL, but it seems likely that its role is exacerbating.

The hemophagocytic syndrome is often seen in this patient group due to massive activation of monocytes and macrophages by cytokines released from the neoplastic cells (Zhang et al. 2014; Maakaroun et al. 2010). The syndrome was also seen in our case.

## Conclusions

ANKL remains a challenging disease to diagnose owing to the fast development, rare nature and varying presentation. However, as reported previously, the leukemic cells in this case showed aberrant expression of several cell markers. It has been suggested that immunohistochemistry and EBV-encoded small RNAs (EBER) detection in a bone marrow biopsy provide a reliable diagnostic approach (Soliman et al. 2014). However, as also reported by others, a bone marrow sample might not be easily obtained, and in addition, the bone marrow samples are rarely the first material to reach the para-clinical laboratories. A bone marrow sample was never taken from the patient presented here due to the very rapid deterioration of her condition. The diagnosis was instead made on blood samples in which the leukemic cells were sufficiently represented. To this aim, the flow cytometric profile was of great assistance to the initial morphological assessment. The potential to stain multiple proteins within a few hours makes the technique a valuable alternative to immunohistochemistry in cases were a complicated diagnosis is needed in very short time. Cell type specific markers provided certainty of the cell of origin in a morphologically difficult leukemia.

## Additional file

10.1186/s40064-015-1553-y Complete flow cytometric data. Histograms of all marker expression evaluated by flow cytometry. Some markers were evaluated multiple times in different antibody mixes using the same or different fluorochromes. In these cases, only one plot is depicted here. All major cell populations are shown in relative sizes as follows: neoplastic NK-cells in olive green, normal lymphocytes in red, granulocytes in yellow and monocytes in blue.

## Abbreviations

ANKL: aggressive natural killer cell leukemia
EBV: Ebstein Barr virus
EBER: EBV-encoded small RNAs
TCR: T cell receptor
SSC: side scatter
FSC: forward scatter
LDH: lactate dehydrogenase
CRP: C-reactive protein
HIV: human immunodeficiency virus
Anti-HBc: hepatitis B virus c-antibody
HBsAg: hepatitis B virus s-antigen
HCV: human cytomegalovirus
