# Supplementary figures and images for: Aggressive NK-cell leukemia in a 69 years old Caucasian woman: a case report
Source: Springerplus. 2015 Dec 9;4:763. doi: 10.1186/s40064-015-1553-y (PMC4673078; doi:10.1186/s40064-015-1553-y)

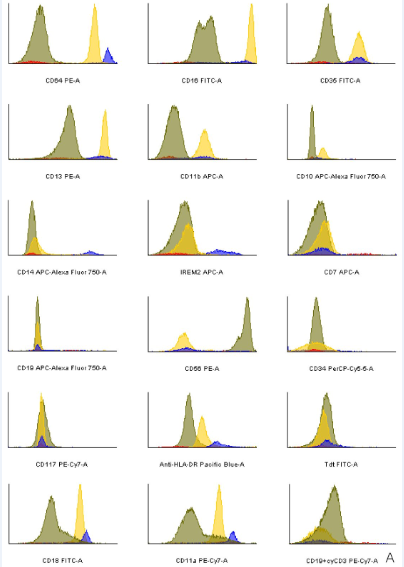


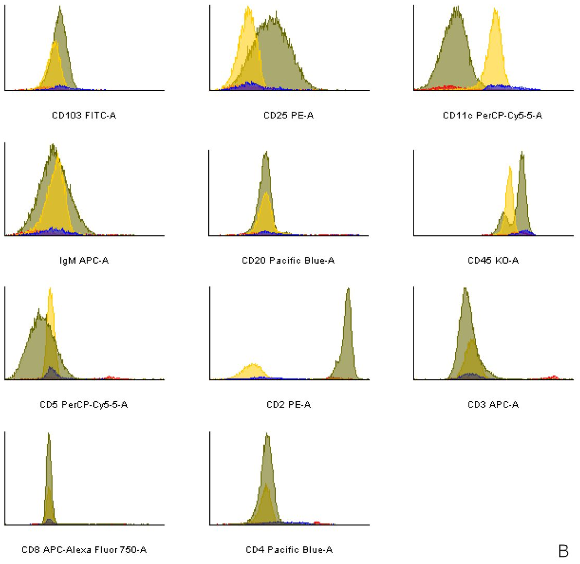

Supplement: Supplementary file 1 — 10.1186/s40064-015-1553-y Complete flow cytometric data. Histograms of all marker expression evaluated by flow cytometry. Some markers were evaluated multiple times in different antibody mixes using the same or different fluorochromes. In these cases, only one plot is depicted here. All major cell populations are shown in relative sizes as follows: neoplastic NK-cells in olive green, normal lymphocytes in red, granulocytes in yellow and monocytes in blue. [file 40064_2015_1553_MOESM1_ESM.docx]
